# Coronary Computed Tomographic Angiography to Optimize the Diagnostic Yield of Invasive Angiography for Low-Risk Patients Screened With Artificial Intelligence: Protocol for the CarDIA-AI Randomized Controlled Trial

**DOI:** 10.2196/71726

**Published:** 2025-05-21

**Authors:** Jeremy Petch, Juan Pablo Tabja Bortesi, Tej Sheth, Madhu Natarajan, Natalia Pinilla-Echeverri, Shuang Di, Shrikant I Bangdiwala, Karen Mosleh, Omar Ibrahim, Kevin R Bainey, Julian Dobranowski, Maria P Becerra, Katie Sonier, Jon-David Schwalm

**Affiliations:** 1 Population Health Research Institute Hamilton, ON Canada; 2 Division of Cardiology Department of Medicine McMaster University Hamilton, ON Canada; 3 Centre for Data Science and Digital Health Hamilton Health Sciences Hamilton, ON Canada; 4 Institute for Health Policy, Management and Evaluation University of Toronto Toronto, ON Canada; 5 School of Epidemiology and Public Health University of Ottawa Ottawa, ON Canada; 6 Dalla Lana School of Public Health University of Toronto Toronto, ON Canada; 7 Department of Health Research Methods, Evidence, and Impact McMaster University Hamilton, ON Canada; 8 Centre for Evidence-Based Implementation Hamilton Health Sciences Hamilton, ON Canada; 9 Mazankowski Alberta Heart Institute University of Alberta Edmonton, AB Canada; 10 Department of Medical Imaging McMaster University Hamilton, ON Canada; 11 Centre for Integrated and Advanced Medical Imaging McMaster University Hamilton, ON Canada; 12 Niagara Health System Saint Catharines, ON Canada

**Keywords:** artificial intelligence, coronary artery disease, coronary computed tomographic angiography, clinical decision support, invasive coronary angiography

## Abstract

**Background:**

Invasive coronary angiography (ICA) is the gold standard in the diagnosis of coronary artery disease (CAD). Being invasive, it carries rare but serious risks including myocardial infarction, stroke, major bleeding, and death. A large proportion of elective outpatients undergoing ICA have nonobstructive CAD, highlighting the suboptimal use of this test. Coronary computed tomographic angiography (CCTA) is a noninvasive option that provides similar information with less risk and is recommended as a first-line test for patients with low-to-intermediate risk of CAD. Leveraging artificial intelligence (AI) to appropriately direct patients to ICA or CCTA based on the predicted probability of disease may improve the efficiency and safety of diagnostic pathways.

**Objective:**

he CarDIA-AI (Coronary computed tomographic angiography to optimize the Diagnostic yield of Invasive Angiography for low-risk patients screened with Artificial Intelligence) study aims to evaluate whether AI-based risk assessment for obstructive CAD implemented within a centralized triage process can optimize the use of ICA in outpatients referred for nonurgent ICA.

**Methods:**

CarDIA-AI is a pragmatic, open-label, superior randomized controlled trial involving 2 Canadian cardiac centers. A total of 252 adults referred for elective outpatient ICA will be randomized 1:1 to usual care (directly proceeding to ICA) or to triage using an AI-based decision support tool. The AI-based decision support tool was developed using referral information from over 37,000 patients and uses a light gradient boosting machine model to predict the probability of obstructive CAD based on 42 clinically relevant predictors, including patient referral information, demographic characteristics, risk factors, and medical history. Participants in the intervention arm will have their ICA referral forms and medical charts reviewed, and select details entered into the decision support tool, which recommends CCTA or ICA based on the patient’s predicted probability of obstructive CAD. All patients will receive the selected imaging modality within 6 weeks of referral and will be subsequently followed for 90 days. The primary outcome is the proportion of normal or nonobstructive CAD diagnosed via ICA and will be assessed using a 2-sided *z* test to compare the patients referred for cardiac investigation with normal or nonobstructive CAD diagnosed through ICA between the intervention and control groups. Secondary outcomes include the number of angiograms avoided and the diagnostic yield of ICA.

**Results:**

Recruitment began on January 9, 2025, and is expected to conclude in mid to late 2025. As of April 14, 2025, we have enrolled 81 participants. Data analysis will begin once data collection is completed. We expect to submit the results for publication in 2026.

**Conclusions:**

CarDIA-AI will be the first randomized controlled trial using AI to optimize patient selection for CCTA versus ICA, potentially improving diagnostic efficiency, avoiding unnecessary complications of ICA, and improving health care resource usage.

**Trial Registration:**

ClinicalTrials.gov NCT06648239; https://clinicaltrials.gov/study/NCT06648239/

**International Registered Report Identifier (IRRID):**

DERR1-10.2196/71726

## Introduction

Coronary artery disease (CAD) is a leading cause of death globally [1]. While invasive coronary angiography (ICA) remains the gold standard for CAD diagnosis, many patients undergoing elective ICA are found to have a nonobstructive disease or normal coronary anatomy, with variability between cardiac centers [2,3]. Despite lower population-based rates of ICA in Ontario, Canada, over 50% of elective ICA procedures find nonsignificant CAD (CorHealth Ontario, unpublished data, 2022). A low diagnostic yield of elective ICA is not only resource-intensive [4-6] but unnecessarily subjects patients not requiring invasive intervention to major procedural complications, including vascular injury, major bleeding, stroke, myocardial infarction, and death [7]. A noninvasive test able to provide similar information with less risk and cost is ideal in a lower-risk population.

Coronary computed tomographic angiography (CCTA) has emerged as one such noninvasive alternative to anatomically evaluate the coronary arteries. CCTA is highly sensitive for the detection of >50% coronary stenosis in patients with low to moderate pretest probability of obstructive CAD [8,9], is less expensive, and carries significantly less risk [10]. Appropriately, guidelines for the evaluation of chest pain from the American College of Cardiology/American Heart Association and the European Society of Cardiology both recommend first-line CCTA in this population [11,12]. However, limited access to CCTA in Canada due to limited diagnostic infrastructures has restricted its widespread use. Conversely, a CCTA-first approach can result in double-testing, since patients with abnormal CCTA frequently proceed to confirmatory or therapeutic ICA, which is wasteful and exposes patients to unnecessary radiation. Ultimately, appropriate test selection requires accurate prediction of the pretest probability of obstructive CAD.

Artificial intelligence (AI) has been leveraged to improve the prediction of significant CAD. Traditional risk assessment tools include a more limited number of variables and may not capture the full complexity of patient risk profiles. Conversely, machine learning algorithms can incorporate a greater number of predictors, including highly correlated predictors, and can account for complex, nonlinear relationships between predictors, including at the patient level [13]. We have developed an AI model to predict the probability of obstructive CAD using 12 years of referral data from 2 regional hospitals, comprising a cohort of over 29,000 patients, which significantly outperformed existing risk scores (area under the receiver operating characteristic curve of 0.81 vs 0.62) and achieved a net reclassification improvement of 44.7% over usual care (95% CI 42.4%-47.0%) [14]. The model has been updated using more recent referral data and has undergone external validation using 2008-2023 province-wide data to confirm generalizability (Petch et al, unpublished paper, April 2025). The integration of this model into clinical practice may enable a more accurate assessment of patient risk and improve diagnostic efficiencies.

**Figure 1:**
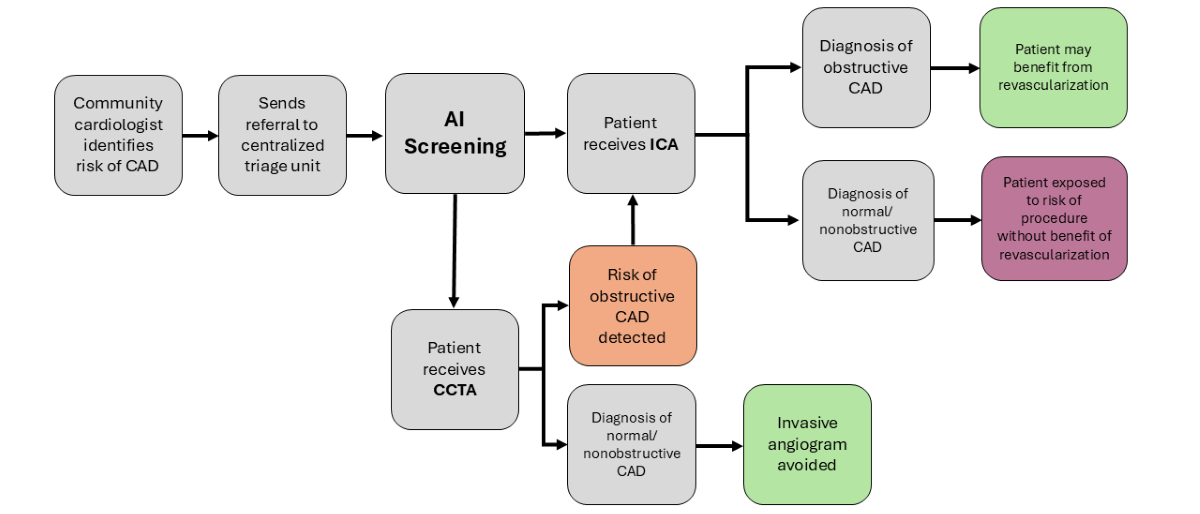
AI intervention in the context of the clinical pathway. The AI model is integrated into a centralized triage model for ICA and CCTA, where community cardiologists can refer to a single unit for an angiogram for suspected obstructive CAD, and the AI model provides a recommendation for which test is most appropriate for a given patient. Green boxes indicate the optimal test for a given patient, orange indicates suboptimal testing (patient received a “double-test” of both CCTA and ICA), and red indicates the least optimal (patient received an ICA but did not have obstructive disease). AI: artificial intelligence; CAD: coronary artery disease; CCTA: coronary computed tomographic angiography; ICA: invasive coronary angiography.

A decision support tool that incorporates such an AI model within a centralized triage pathway may facilitate patient selection for first-line CCTA (Figure 1). We have established that a centralized triage model in which low-risk patients referred for ICA are considered for CCTA first can improve the diagnostic yield of elective ICA and that such a model is acceptable to both patients and referring physicians [15]. As such, we have deployed the AI model in our local cardiac center and conducted a pilot study using a silent trial design [16] to establish feasibility within the centralized triage pathway and address implementation issues (Schwalm et al, unpublished paper, April 2025). With the individual components of the intervention validated and piloted, the next appropriate step is to conduct a clinical trial of the intervention to measure its impact on clinical outcomes.

The CarDIA-AI (Coronary computed tomographic angiography to optimize the Diagnostic yield of Invasive Angiography for low-risk patients screened with Artificial Intelligence) trial will evaluate whether AI-based screening within a centralized triage pathway can optimize patient selection for CCTA versus ICA. The primary goal of this study is to reduce the proportion of unnecessary ICA procedures (showing normal or nonobstructive CAD) performed in patients referred for evaluation of CAD diagnosed in patients undergoing ICA. This trial will compare the AI intervention to usual care (ie, directly proceeding to ICA) to assess improvements beyond the current diagnostic pathway.

## Methods

### Objectives

The primary objective of the CarDIA-AI trial is to determine the effect of a centralized triage process incorporating AI-based risk assessment, compared to usual care, on the proportion of normal or nonobstructive CAD diagnosed via ICA, in adults referred for nonurgent (elective) outpatient ICA.

### Study Design

The CarDIA-AI trial is a pragmatic, open-label, multicenter, superiority trial with two parallel groups and 1:1 allocation. The study was registered at ClinicalTrials.gov (NCT06648239). This protocol was prepared in accordance with the SPIRIT-AI (Standard Protocol Items: Recommendations for Interventional Trials—Artificial Intelligence) guidelines [17] (see Multimedia Appendix 1 for checklist).

### Participants

#### Setting

Two cardiac centers located in Ontario, Canada, will participate in the CarDIA-AI trial: Hamilton Health Sciences’ General Hospital (Hamilton site) and Niagara Health System’s Marotta Family Hospital (Niagara site). These regional referral centers serve a large geographic area in southern Ontario with a population exceeding 1.8 million. ICA referral forms from the region are received at the Heart Investigation Unit located at the Hamilton site. Ordinarily, triage staff categorize the referrals according to urgency and site (Hamilton or Niagara; based on the patient’s home address). For CarDIA-AI, outpatient ICA referrals will be reviewed daily to identify eligible participants.

The AI risk-assessment tool is deployed on a virtual machine housed at Hamilton Health Sciences’ research data center. The virtual machine is allocated with 8 central processing unit cores and 16 GB of RAM. The virtual machine is accessed by the research team at the study sites using Citrix.

#### Informed Consent

Eligible patients are identified by a research assistant and reviewed by a board-certified cardiologist. The research assistant will then contact eligible patients by phone to discuss the study, review the consent form, and obtain verbal informed consent. At the time of the ICA or CCTA, research staff will also obtain written, paper-based consent from patients willing to participate (Multimedia Appendix 2).

#### Sample Size

The assumed proportion of normal or nonobstructive CAD on angiogram in the control arm is based on proportions at the two participating centers over the last decade, which have been stable at 44%. Our pilot work suggests that the proportion of normal or nonobstructive CAD diagnosed via ICA in the experimental arm is expected to be around 22%. We studied the power for various possible effect sizes (reduction in event proportions). A 1:1 sample of 200 people can detect an absolute reduction of 22% or higher in event proportion with 91.9% power, and a smaller 19% absolute reduction or higher with 81.3% power. To adjust for noncompliance of up to 5% in both arms, we increased the sample size from 200 patients by a factor of 1.23 to 246 patients [18]. Adjusting for up to 2% losses to follow-up, we require 252 patients (126 per arm).

An average of 115 eligible patients are seen at the participating centers each month. Given that recruitment is carried out by a centralized administration team (individual clinicians are not required to be involved), we do not anticipate significant recruitment barriers. Thus, we conservatively expect to recruit 100 patients per month. The recruitment will take place over 3 months to achieve our target sample size.

#### Eligibility Criteria

Patients are eligible if they (1) are 18 years of age or older; (2) are referred for nonurgent (elective) outpatient ICA; (3) have an indication for ICA that includes “Rule out CAD,” “Cardiomyopathy,” “Stable CAD,” or “Stable Angina”; and (4) are able to provide informed consent in English. Patients will be excluded if they (1) have received a prior high-quality CCTA within the last five years; (2) have atrial fibrillation or other arrhythmia precluding high-quality CCTA; (3) have known severe renal dysfunction (glomerular filtration rate of <35 mL/min/1.73 m^2^); (4) have planned (coronary or noncoronary) cardiac surgery; (5) have any prior obstructive CAD, acute coronary syndrome, percutaneous coronary intervention, or coronary artery bypass graft; or (6) have known severe coronary artery calcification (calcium score >250) or not deemed to be “mild” on report if not quantified. Referral forms indicating “Stable CAD” will be reviewed to ensure the patient does not meet exclusion criteria (eg, known prior obstructive CAD), as many referring physicians check this indication without objective evidence of known CAD.

No exclusions will be made at the level of the input data after confirming patient-level eligibility. If data on the referral form are missing or of poor quality, research staff will review any additional documentation in the patient’s chart to attempt to obtain the missing data. Otherwise, the data will be imputed.

#### Randomization

Participants will be randomly assigned (1:1 allocation) to either usual care or centralized triage using AI-based CAD risk assessment. Block randomization using randomly varying block sizes (unknown to investigators and study personnel), stratified by site with a computer-generated random sequence, will be used. Research personnel will randomize participants via an interactive web response system, ensuring allocation concealment. The interactive web response system is a 24-hour computerized randomization internet system maintained by the coordinating center at Hamilton Health Sciences’ Population Health Research Institute.

### Interventions

#### Centralized Triage

**Figure 2:**
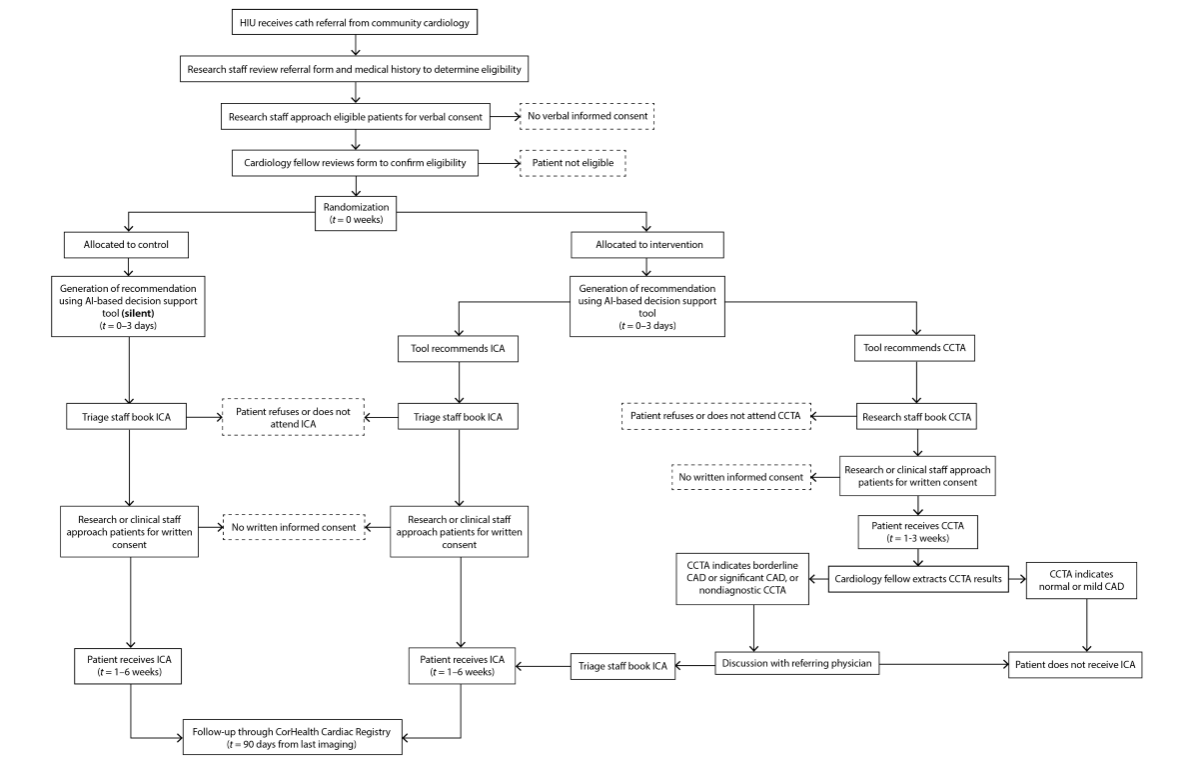
The flow of participants through the study. AI: artificial intelligence; CAD: coronary artery disease; CCTA: coronary computed tomographic angiography; HIU: Heart Investigation Unit; ICA: invasive coronary angiography.

Participants randomized to the intervention arm will have their referral triaged using an AI-based decision support tool to determine the cardiac imaging test they will receive. The AI model uses participants’ medical history (as documented on their referral form) to predict the patient’s probability of significant CAD. Participants deemed high risk for obstructive CAD will receive ICA as recommended by the AI model, effectively receiving usual care. Those assessed to be low risk will receive CCTA, proceeding to confirmatory or therapeutic ICA if appropriate (Figure 2). For patients undergoing CCTA, recommendations for medical management versus referral for ICA will be made based on the CCTA results as per established clinical practice guidelines (Table S1 in Multimedia Appendix 3). Participants who withdraw consent or are admitted to the hospital with concern for acute coronary syndrome after randomization will receive usual care. As our pilot study suggests that the centralized triage process is acceptable and has high fidelity [15], no specific strategies to improve intervention adherence will be implemented.

Only participants in the intervention arm will receive CCTA. Furthermore, interventional cardiologists require access to CCTA results to provide the best care and determine the appropriateness of subsequent ICA. As such, blinding of patients and providers is not possible.

#### AI-Based Risk Assessment for Significant CAD

As part of the centralized triage process, participants’ allocation status will be entered into the risk assessment tool prior to generating the recommendation. For patients randomized to the intervention, the AI model outputs a predicted probability of significant CAD (not shared with clinical personnel), as well as an imaging modality recommendation (CCTA or ICA) informed by the predicted probability. Participants in the intervention arm will receive the imaging modality recommended by the tool.

The AI model uses a light gradient boosting machine algorithm to predict the probability of obstructive CAD based on 42 clinical predictors, including demographic characteristics, patient referral information, anthropometric measures, clinical symptoms, risk factors, medical history, and tobacco habits, as documented on the referral forms (Multimedia Appendix 4). Based on clinical input, we included variables related to the patient’s medical history available at the time of referral, the unique physician number for each referring physician to capture patterns in referral practices, and socioeconomic variables. We selected the light gradient boosting machine algorithm based on our previous study, in which it showed the best discrimination performance among 8 machine learning models developed [14]. An analysis of variable importance and interactions between variables can be found in our previous publication [14]. The model was trained, validated, and evaluated on data from the CorHealth Ontario Cardiac Registry, a multicenter, prospectively collected registry of referral information from patients receiving ICA in the province of Ontario, Canada. The original model derivation cohort included 29,448 patients who received their ICA in the Hamilton or Niagara regions between 2008 and 2019 [14]. We subsequently externally validated the model using an additional sample of 7785 patients who underwent ICA between 2020 and 2023. This trial will use the most recent version of the model, based on 2008-2023 data from the two trial sites. The decision thresholds are 0.556 and 0.560 for the Hamilton and Niagara sites, respectively. The thresholds were optimized to maximize the difference between the true positive rate and the false positive rate (Youden index). An exhaustive description of the development, validation, and evaluation methods and results for this model will be available in a forthcoming paper (Petch et al, unpublished paper, April 2025). The AI model is not currently publicly available.

To obtain the input data for the tool, a research assistant will manually review the ICA referral forms of eligible patients and associated documentation in their chart (Figure S1 in Multimedia Appendix 3). If there are ambiguities or inconsistencies in the referral form or chart, these data will be managed based on predetermined standard operating procedures (Multimedia Appendix 5). Missing data will be imputed. Any ambiguities or inconsistencies requiring clinical interpretation will be treated as missing and imputed.

The user interface of the AI tool consists of various input fields that correspond to the model predictors (Figure S2 in Multimedia Appendix 3). Users either manually enter a value (eg, for Age) or select a value from the field drop-down menu (eg, for Referral Month). The input fields largely reflect nominal, structured data elements on the referral forms completed by referring physicians. While clinical expertise is not required to use the tool, study personnel using the tool have discussed the contents of the form at length with members of the Heart Investigation Unit in preparation for the trial.

#### Usual Care

Participants allocated to the control arm will receive usual care, meaning they proceed directly to ICA as referred. For control group participants, the AI-based decision support tool will always recommend ICA (ie, usual care) and provide the predicted probability of significant CAD, though this probability will not be shared with the clinical team.

### Outcomes

#### Primary Outcome

The primary outcome is the proportion of normal or nonobstructive CAD diagnosed via ICA, calculated by dividing the number of patients diagnosed with normal or nonobstructive CAD through ICA by the total patients allocated to that arm. This was identified as the most policy-relevant outcome for Ontario Health.

#### Secondary Outcomes

Secondary outcomes include (1) the number of angiograms avoided, (2) deviation from management recommendations following CCTA (ie, angiograms performed when not recommended), (3) the diagnostic yield of ICA (ie, number of patients diagnosed with obstructive CAD out of all patients undergoing ICA), (4) the number of low-quality CCTAs, (5) the difference in the proportion of normal or nonobstructive CAD diagnosed through ICA between male and female patients; and (6) the difference in the proportion of normal or nonobstructive CAD diagnosed through ICA between sites. Furthermore, we will assess the budget impact of the AI intervention.

### Data Collection

#### Overview

All patients will be followed for 90 days from the date of the final diagnostic procedure to capture any relevant outcomes in this stable, elective outpatient population. For patients receiving only ICA or CCTA, this would be the date of the diagnostic procedure; for patients receiving ICA after an initial CCTA, this would be the date of the ICA. All patients will have a CCTA or ICA within 6 weeks of their index referral as per provincial guidelines. Outcomes will be identified at the time one or both procedures (if indicated) are complete. We are allowing an additional 90 days from the final diagnostic procedure to capture any unplanned crossover from CCTA to ICA (see Safety). Follow-up will be done through the provincial CorHealth Ontario Cardiac Registry, housed at the Institute for Clinical Evaluative Sciences. All ICA findings in Ontario are transmitted to the provincial registry via its secure portal. We will extract ICA findings from the registry and derive our outcomes using the standard definition for normal or nonsignificant CAD: <70% stenosis on a major coronary vessel (>2 mm) or <50% stenosis on the left main. For patients who receive CCTA, a cardiologist will manually extract CCTA results from their charts as part of follow-up, as these results are not collected by CorHealth. CCTA findings will be collected in a format and structure that aligns with the CorHealth data collection standards used for ICA findings.

For each CCTA, we will assess the quality of the resulting image. The quality will be graded on a per-patient basis using a 3-class system: low quality, denoting an image in which the coronary anatomy cannot be clearly defined, requiring ICA within 90 days for clarification; suboptimal quality, denoting an image in which the coronary anatomy was equivocal for one or more nonprognostic vessels but not requiring ICA based on CCTA findings and clinical presentation; and high quality, denoting an image in which the coronary anatomy could be clearly defined.

To track participant retention, we will keep a log of all included participants to document any who are lost to follow-up, withdraw consent, or do not otherwise receive the recommended imaging intervention. Additionally, we will document all reasons for withdrawal, as well as ask whether the patient intends to proceed with usual care (if the patient is withdrawing prior to their imaging procedure). As the necessary outcome data is generated at the time of CCTA or ICA and direct participant involvement is not required for follow-up, the participant burden will be minimized.

#### Implementation Study

As part of a parallel implementation study, we will identify anticipated challenges to the implementation of the centralized triage process with AI-based risk assessment for CAD. Semistructured interviews and electronic surveys (via REDCap [Research Electronic Data Capture]; Vanderbilt University) will be conducted with key stakeholders—including the catheterization laboratory director, catheterization laboratory manager, catheterization laboratory triage staff, and representation from the CCTA program—at 3 cardiac centers not participating in CarDIA-AI. Survey and interview guides will explore a range of stakeholder perspectives including their awareness of the innovation and attitudes toward the change generally, as well as specific to the innovation, any skills and experiences they might have that could be required in the implementation of the innovation, any concerns about the proposed change, and their intentions to adopt or use the innovation. Development of the survey and interview questions will be informed by the updated Consolidated Framework for Implementation Research, a determinant framework to explain barriers and facilitators to implementation effectiveness [19]. At the participating centers, interviews will be conducted with triage staff to assess their perceptions of the AI model’s success and identify what would be needed to integrate it completely into processes at their site. A list of potential interview and survey questions is available in Multimedia Appendix 6. Descriptive analysis of quantitative survey results (eg, Likert scale responses) will be conducted by the research team. Qualitative analysis of open-ended survey questions and interview transcripts will be carried out using thematic analysis approaches via NVivo software (Lumivero). Data will be coded and mapped to selected constructs of the Consolidated Framework for Implementation Research. Findings will be used to inform the development of strategies and interventions to mitigate identified barriers to the implementation of the centralized triage process if it is determined to be effective. Key barriers will be mapped to the most appropriate implementation strategies from an established compilation of potential strategies, the Expert Recommendations for Implementing Change [20].

### Analyses

#### Statistical Analysis

The primary analysis will compare the proportion of patients referred for cardiac investigation with normal or nonobstructive CAD diagnosed through ICA between the intervention and control groups with a 2-sided *z* test comparing two proportions. All analyses will be carried out according to the intention-to-treat principle; that is, all participants with complete data will be analyzed according to the group to which they were randomized, including noncompliant participants. There are no planned interim analyses. A table of baseline characteristics will summarize patients overall and by randomization arm. Continuous variables will be summarized as mean (SD) or median (IQR), with comparisons between randomization arms made by a 2-sample *t* test (or Wilcoxon rank-sum test if data are not normally distributed). Categorical variables will be summarized as count and percentage, with comparison using the Pearson chi-square test. All statistical analyses will be performed using SAS software (version 9.4; SAS Institute Inc). We will report *P* values up to 3 decimal places and report *P* values less than .001 as <.001. We will use a type I error level of 5%.

We expect a small amount of missing data due to noncompliance and loss to follow-up. As noted above, participants who withdraw will be asked for a reason for withdrawal to determine whether the missing information is random. Participants withdrawing prior to the procedure will be asked whether they intend to proceed to ICA. Finally, we intend to conduct sensitivity analyses based on best- and worst-case scenarios to assess the impact of missing data on the results.

As part of our secondary outcome analysis, we plan to examine whether the intervention can address disparities in ICA findings and evaluate its effects across different sites and eligibility criteria. We will stratify by sex to evaluate whether the intervention reduces the discrepancy in the proportion of normal findings on ICA between male and female patients (female patients are currently 40% more likely to have a normal finding on ICA, meaning female patients are less likely than male patients to experience the therapeutic benefits associated with ICA). Similarly, we also plan to stratify by site to evaluate whether there are differences in the intervention effects between the Hamilton and Niagara sites. To simulate the impacts of using different criteria for CCTA-first eligibility, we will also evaluate the sensitivity of all outcomes to changes to the exclusion criteria, such as incorporating age- and sex-related exclusions. Formal statistical tests will not be used, as we do not anticipate that these hypothesis-generating secondary analyses will be adequately powered.

Given our relatively small sample size, there may be an imbalance at baseline for important covariates. We will therefore undertake an adjusted analysis as a secondary sensitivity analysis, adjusting for age and sex. These covariates were selected based on our external validation study (Petch et al, unpublished paper, April 2025), which found an association between these covariates and the outcome (event rate for male individuals was 71% vs 53% for female individuals, and 68% for those older than 65 years vs 59% for those 65 years and younger).

#### Economic Analysis

For the budget impact analysis, we will use the trial data and Ontario Health Insurance Program professional and technical fees to compare the procedural costs of screening with usual care (cost of ICA) and the AI model (weighted average of the cost of ICA and the cost of CCTA based on the AI model recommendation and CCTA output). A catheterization laboratory cost, which is not included in Ontario Health Insurance Program fees, will be added to the cost of ICA. In a secondary economic analysis, the 90-day health care expenditures post index investigation (eg, revascularization and hospitalization) will be derived using the Institute for Clinical Evaluative Science databases and compared between the 2 diagnostic strategies. Bootstrap techniques will be used to deal with sampling uncertainty and calculate 95% CIs around the costs [21]. The per-patient costs will be extrapolated to Ontario and Canada to document the health care cost savings at the population level.

### Safety

Both CCTA and ICA are considered standard of care in the patient population for CarDIA-AI. A total of 50% (126/252) of patients (control arm) will automatically receive usual care. Patients randomized to the intervention who receive a recommendation to proceed directly to ICA will effectively also receive usual care. Patients who receive a recommendation to proceed to CCTA will not be at increased risk of missed diagnosis, as CCTA has been found to be as effective as ICA in both diagnosing and ruling out obstructive CAD in stable outpatients [9,22-24]. A potential harm in the intervention group is double testing, which would occur when the CCTA is of poor quality and the degree of obstructive CAD cannot be clearly quantified. Double testing may also occur when the AI model predicts a low probability of obstructive CAD, and in turn, recommends CCTA, but the CCTA is positive and the patient requires confirmatory or therapeutic ICA. Both of these potential harms are only expected to occur in a small number of patients, and the risk in the intervention group will be much lower as compared to usual care. We will also track ICA within 90 days of the last imaging procedure to ensure that there are no events in the intervention group representing unplanned crossover.

In terms of potential model performance errors, the data quality committee will periodically monitor the performance of the model, with particular attention to when the model appears to be performing outside its normal parameters. No formal auditing is planned.

### Ethical Considerations

This study was approved by the Hamilton Integrated Research Ethics Board (HiREB #17103, protocol version 1.8, February 21, 2025. The original study protocol was first approved on November 5, 2024). At the time of recruitment, participants will be provided with the informed consent form by email and will be given the opportunity to ask questions about the study. The email sent to participants contains the contact information of two members of the research team, as well as instructions for participants should they have further questions about the study or wish to opt out. Documented consent will be obtained either before or at the time of the index procedure (CCTA or ICA). Participants are made aware during the consent process that they will not be provided monetary compensation for participating in the study. The data obtained from the referral forms and medical histories that are entered into the decision support tool, along with the predicted probability of significant CAD, will be automatically stored in a secure, cloud-based database upon generation of the recommendation, with the exception of postal codes and referring physicians’ College of Physicians and Surgeons of Ontario registration numbers. Participant data will be handled in compliance with all relevant privacy and security regulations. All personal information about potential and enrolled participants will be securely stored and accessible only to authorized research team members. To protect confidentiality, participants will be assigned unique identification numbers, which will link their personal data to study-related data. All study data will be deidentified before being stored in the study database, which is maintained on a secure institutional server with restricted access.

## Results

CarDIA-AI received funding in 2023. Recruitment began on January 9, 2025, and is projected to conclude in mid to late 2025. As of April 14, 2025, we have enrolled 81 participants. Data analysis will begin after all follow-up data are obtained. We expect to submit the first results for publication in 2026.

## Discussion

### Contributions of the CarDIA-AI Trial

Appropriate test selection for evaluating the coronary anatomy of elective outpatients with suspected CAD is important for reducing unnecessary invasive procedures and optimizing health care resource usage. A recent statement from various radiology and cardiovascular imaging societies called attention to the need for research on the use of AI for personalized cardiac imaging test selection [25]. The CarDIA-AI trial aims to meet this need as the first to leverage AI to optimize patient selection for CCTA versus ICA. Building on strong pilot work demonstrating the feasibility and potential of the AI model and triage pathway, this trial will evaluate their effectiveness in clinical practice. Based on our previous work [14], we anticipate that patients triaged using the AI-based decision support tool will have a lower proportion of normal or nonobstructive CAD diagnosed via ICA compared to those proceeding directly to ICA. If successful, this intervention could streamline the triage of ICA referrals and enhance the use of CCTA, which could save health care systems millions of dollars each year while preventing procedural complications.

### Comparison to Prior Work

In the assessment of stable CAD, various prediction models have been developed to estimate the probability of disease prior to diagnostic evaluation [26,27]. While most validated models rely on traditional statistical approaches, there is growing interest in the application of AI methods. In other areas of cardiology, AI has recently been successfully implemented to support the triage of chest pain in emergency care settings, reducing time to emergency cardiovascular procedures [28,29]. Another recent trial evaluated an AI model that predicts the probability of significant CAD from stress echocardiography images to guide referrals for ICA [30], highlighting the increasing focus on AI in cardiology decision-making. However, while AI models have been developed for numerous use cases in cardiology, their evaluation in clinical trials has been relatively limited thus far. Most models that have been prospectively assessed have demonstrated clinical or operational improvements relative to clinicians, particularly in applications related to imaging interpretation and CAD [31]. Despite these promising findings, there is still a need for prospective trials to determine the real-world effectiveness of AI interventions [32]. The CarDIA-AI trial will address this gap by evaluating an AI model in a pragmatic manner and inform future implementation strategies for similar interventions.

### Strengths and Limitations

There are some limitations to our approach. Based on our pilot studies, we expect a certain amount of data to be missing from some patients’ referral forms, which we have found to have a small effect on model performance compared to our original retrospective study. However, while our model may achieve slighter lower predictive performance than it would using retrospective data, the pragmatic nature of our trial design should ultimately yield a more realistic estimate of the effects this AI model would achieve in a real-world setting. Similarly, the immediate generalizability of the AI model remains uncertain, as its effectiveness may vary across different health care settings. However, the parallel implementation study will evaluate how effectively the intervention can be integrated into practice, identifying barriers and facilitators to clarify the contexts in which such an intervention may be most effective. Additionally, while the AI model is specific to the Ontario data on which it was developed and validated, similar datasets exist in other jurisdictions. While we are not assessing clinical efficacy and safety outcomes in this trial, these have been previously established in trials comparing CCTA-first strategies to direct ICA [33-36].

### Future Directions

Further research is needed to more comprehensively understand the potential of the AI intervention and its sustainability. For example, prospective evaluations in other centers will provide insight into its generalizability and scalability. The implementation study will qualitatively explore barriers to using the intervention as perceived by triage staff. Future studies where the tool is actively used by triage staff should be conducted to further identify potential usability challenges and assess whether the intervention remains effective in routine practice. Similarly, future work may also explore the long-term effectiveness of the AI intervention, for example, by implementing methods to continually monitor model performance.

### Conclusions

The CarDIA-AI trial is the first to leverage AI for the triage of patients referred for assessment of suspected CAD. If successful, the intervention could optimize the referral process, resulting in millions of dollars in cost savings to the health care system while improving the patient experience by reducing exposure to unnecessary procedural risks.

## Appendix

Multimedia Appendix 1 SPIRIT-AI (Standard Protocol Items: Recommendations for Interventional Trials—Artificial Intelligence) checklist.

Multimedia Appendix 2 Informed consent form.

Multimedia Appendix 3 Additional tables and figures.

Multimedia Appendix 4 Model input details.

Multimedia Appendix 5 Data entry standard operating procedure.

Multimedia Appendix 6 CarDIA-AI (CCTA [coronary computer tomographic angiography] to optimize the Diagnostic yield of Invasive Angiography for low-risk patients screened with AI [artificial intelligence]) implementation study interview and survey questions.

Multimedia Appendix 7 Information on trial sponsor, steering committee, and data quality committee.

## Abbreviations

AI: artificial intelligence
CAD: coronary artery disease
CarDIA-AI: CCTA to optimize the Diagnostic yield of Invasive Angiography for low-risk patients screened with AI
CCTA: coronary computed tomographic angiography
ICA: invasive coronary angiography
REDCap: Research Electronic Data Capture
SPIRIT-AI: Standard Protocol Items: Recommendations for Interventional Trials—Artificial Intelligence

## References

[1] Nowbar AN, Gitto M, Howard JP, Francis DP, Al-Lamee R (2019). Mortality from ischemic heart disease. Circ Cardiovasc Qual Outcomes.

[2] Patel MR, Peterson ED, Dai D, Brennan JM, Redberg RF, Anderson HV, Brindis RG, Douglas PS (2010). Low diagnostic yield of elective coronary angiography. N Engl J Med.

[3] Bradley SM, Maddox TM, Stanislawski MA, O'Donnell CI, Grunwald GK, Tsai TT, Ho PM, Peterson ED, Rumsfeld JS (2014). Normal coronary rates for elective angiography in the veterans affairs healthcare system: insights from the VA CART program (veterans affairs clinical assessment reporting and tracking). J Am Coll Cardiol.

[4] (2010). The relative cost-effectiveness of five non-invasive cardiac imaging technologies for diagnosing coronary artery disease in Ontario. Toronto Health Economics and Technology Assessment Collaborative.

[5] Oseran AS, Ati S, Feldman WB, Gondi S, Yeh RW, Wadhera RK (2022). Assessment of prices for cardiovascular tests and procedures at top-ranked US hospitals. JAMA Intern Med.

[6] Darlington M, Gueret P, Laissy JP, Pierucci AF, Maoulida H, Quelen C, Niarra R, Chatellier G, Durand-Zaleski I (2015). Cost-effectiveness of computed tomography coronary angiography versus conventional invasive coronary angiography. Eur J Health Econ.

[7] Tavakol M, Ashraf S, Brener SJ (2012). Risks and complications of coronary angiography: a comprehensive review. Global J Health Sci.

[8] Haase R, Schlattmann P, Gueret P, Andreini D, Pontone G, Alkadhi H, Hausleiter J, Garcia MJ, Leschka S, Meijboom WB, Zimmermann E, Gerber B, Schoepf UJ, Shabestari AA, Nørgaard BL, Meijs MFL, Sato A, Ovrehus KA, Diederichsen ACP, Jenkins SMM, Knuuti J, Hamdan A, Halvorsen BA, Mendoza-Rodriguez V, Rochitte CE, Rixe J, Wan YL, Langer C, Bettencourt N, Martuscelli E, Ghostine S, Buechel RR, Nikolaou K, Mickley H, Yang L, Zhang Z, Chen MY, Halon DA, Rief M, Sun K, Hirt-Moch B, Niinuma H, Marcus RP, Muraglia S, Jakamy R, Chow BJ, Kaufmann PA, Tardif J, Nomura C, Kofoed KF, Laissy J, Arbab-Zadeh A, Kitagawa K, Laham R, Jinzaki M, Hoe J, Rybicki FJ, Scholte A, Paul N, Tan SY, Yoshioka K, Röhle R, Schuetz GM, Schueler S, Coenen MH, Wieske V, Achenbach S, Budoff MJ, Laule M, Newby DE, Dewey M (2019). Diagnosis of obstructive coronary artery disease using computed tomography angiography in patients with stable chest pain depending on clinical probability and in clinically important subgroups: meta-analysis of individual patient data. BMJ.

[9] Sheth T, Amlani S, Ellins ML, Mehta S, Velianou J, Cappelli G, Yang S, Natarajan M (2008). Computed tomographic coronary angiographic assessment of high-risk coronary anatomy in patients with suspected coronary artery disease and intermediate pretest probability. Am Heart J.

[10] Mancini GBJ, Leipsic J, Budoff MJ, Hague CJ, Min JK, Stevens SR, Reynolds HR, O'Brien SM, Shaw LJ, Manjunath CN, Mavromatis K, Demkow M, Lopez-Sendon JL, Chernavskiy AM, Gosselin G, Schuchlenz H, Devlin GP, Chauhan A, Bangalore S, Hochman JS, Maron DJ (2021). CT Angiography followed by invasive angiography in patients with moderate or severe ischemia-insights from the ISCHEMIA trial. JACC Cardiovasc Imaging.

[11] Knuuti J, Wijns W, Saraste A, Capodanno D, Barbato E, Funck-Brentano C, Prescott E, Storey RF, Deaton C, Cuisset T, Agewall S, Dickstein K, Edvardsen T, Escaned J, Gersh BJ, Svitil P, Gilard M, Hasdai D, Hatala R, Mahfoud F, Masip J, Muneretto C, Valgimigli M, Achenbach S, Bax JJ (2020). 2019 ESC guidelines for the diagnosis and management of chronic coronary syndromes. Eur Heart J.

[12] Gulati M, Levy PD, Mukherjee D, Amsterdam E, Bhatt DL, Birtcher KK, Blankstein R, Boyd J, Bullock-Palmer RP, Conejo T, Diercks DB, Gentile F, Greenwood JP, Hess EP, Hollenberg SM, Jaber WA, Jneid H, Joglar JA, Morrow DA, O'Connor RE, Ross MA, Shaw LJ (2021). 2021 AHA/ACC/ASE/CHEST/SAEM/SCCT/SCMR guideline for the evaluation and diagnosis of chest pain: a report of the American College of Cardiology/American Heart Association Joint Committee on clinical practice guidelines. Circulation.

[13] Goldstein BA, Navar AM, Carter RE (2017). Moving beyond regression techniques in cardiovascular risk prediction: applying machine learning to address analytic challenges. Eur Heart J.

[14] Schwalm JD, Di S, Sheth T, Natarajan MK, O'Brien E, McCready T, Petch J (2022). A machine learning-based clinical decision support algorithm for reducing unnecessary coronary angiograms. Cardiovasc Digital Health J.

[15] Schwalm JD, Bouck Z, Natarajan MK, Pinilla N, Walker D, Syed N, Landry D, Sabri A, Tandon V, Nkurunziza J, Taljaard M, Sheth T (2023). Centralized triage of suspected coronary artery disease using coronary computed tomographic angiography to optimize the diagnostic yield of invasive angiography. CJC Open.

[16] Kwong JCC, Erdman L, Khondker A, Skreta M, Goldenberg A, McCradden MD, Lorenzo AJ, Rickard M (2022). The silent trial—the bridge between bench-to-bedside clinical AI applications. Front Digital Health.

[17] Rivera SC, Liu X, Chan AW, Denniston AK, Calvert MJ, The SPIRIT-AI and CONSORT-AI Working Group (2020). Guidelines for clinical trial protocols for interventions involving artificial intelligence: the SPIRIT-AI extension. BMJ.

[18] Wittes J (2002). Sample size calculations for randomized controlled trials. Epidemiol Rev.

[19] Damschroder LJ, Reardon CM, Widerquist MAO, Lowery J (2022). The updated consolidated framework for implementation research based on user feedback. Implement Sci.

[20] Waltz TJ, Powell BJ, Fernández ME, Abadie B, Damschroder LJ (2019). Choosing implementation strategies to address contextual barriers: diversity in recommendations and future directions. Implement Sci.

[21] Efron B, Tibshirani R (1993). An Introduction to the Bootstrap.

[22] Maffei E, Seitun S, Martini C, Palumbo A, Tarantini G, Berti E, Grilli R, Tedeschi C, Messalli G, Guaricci A, Weustink AC, Mollet NR, Cademartiri F (2010). CT coronary angiography and exercise ECG in a population with chest pain and low-to-intermediate pre-test likelihood of coronary artery disease. Heart.

[23] Nielsen LH, Ortner N, Nørgaard BL, Achenbach S, Leipsic J, Abdulla J (2014). The diagnostic accuracy and outcomes after coronary computed tomography angiography vs. conventional functional testing in patients with stable angina pectoris: a systematic review and meta-analysis. Eur Heart J Cardiovasc Imaging.

[24] Budoff MJ, Dowe D, Jollis JG, Gitter M, Sutherland J, Halamert E, Scherer M, Bellinger R, Martin A, Benton R, Delago A, Min JK (2008). Diagnostic performance of 64-multidetector row coronary computed tomographic angiography for evaluation of coronary artery stenosis in individuals without known coronary artery disease: results from the prospective multicenter ACCURACY (Assessment by Coronary Computed Tomographic Angiography of Individuals Undergoing Invasive Coronary Angiography) trial. J Am Coll Cardiol.

[25] Mastrodicasa D, van Assen M, Huisman M, Leiner T, Williamson EE, Nicol ED, Allen BD, Saba L, Vliegenthart R, Hanneman K (2025). Use of AI in cardiac CT and MRI: a scientific statement from the ESCR, EuSoMII, NASCI, SCCT, SCMR, SIIM, and RSNA. Radiology.

[26] He T, Liu X, Xu N, Li Y, Wu Q, Liu M, Yuan H (2017). Diagnostic models of the pre-test probability of stable coronary artery disease: a systematic review. Clinics (Sao Paulo).

[27] Mincarone P, Bodini A, Tumolo MR, Vozzi F, Rocchiccioli S, Pelosi G, Caselli C, Sabina S, Leo CG (2021). Discrimination capability of pretest probability of stable coronary artery disease: a systematic review and meta-analysis suggesting how to improve validation procedures. BMJ Open.

[28] Wang YC, Chen KW, Tsai BY, Wu M, Hsieh P, Wei J, Shih ES, Shiao Y, Hwang M, Chang K (2022). Implementation of an all-day artificial intelligence-based triage system to accelerate door-to-balloon times. Mayo Clin Proc.

[29] Hinson JS, Taylor RA, Venkatesh A, Steinhart BD, Chmura C, Sangal RB, Levin SR (2024). Accelerated chest pain treatment with artificial intelligence-informed, risk-driven triage. JAMA Intern Med.

[30] Upton R, Akerman AP, Marwick TH, Johnson CL, Piotrowska H, Bajre M, Breen M, Dawes H, Dehbi H, Descamps T, Harris V, Hawkes W, Krasner S, Sanderson E, Savage N, Thompson B, Williamson V, Woodward W, Sarwar R, O’Driscoll J, Sharma R, Chiocchia V, Petersen SE, Frangou E, Ridgway G, Bhattacharyya S, Ripley DP, Woodward G, Leeson P (2024). PROTEUS: a prospective RCT evaluating use of AI in stress echocardiography. NEJM AI.

[31] Moosavi A, Huang S, Vahabi M, Motamedivafa B, Tian N, Mahmood R, Liu P, Sun CL (2024). Prospective human validation of artificial intelligence interventions in cardiology: a scoping review. JACC Adv.

[32] Ouyang D, Hogan J (2024). We need more randomized clinical trials of AI. NEJM AI.

[33] The DISCHARGE Trial Group (2022). CT or invasive coronary angiography in stable chest pain. N Engl J Med.

[34] Dewey M, Rief M, Martus P, Kendziora B, Feger S, Dreger H, Priem S, Knebel F, Böhm M, Schlattmann P, Hamm B, Schönenberger E, Laule M, Zimmermann E (2016). Evaluation of computed tomography in patients with atypical angina or chest pain clinically referred for invasive coronary angiography: randomised controlled trial. BMJ.

[35] Rudziński PN, Kruk M, Demkow M, Oleksiak A, Schoepf JU, Mach M, Dzielińska Z, Pręgowski J, Witkowski A, Rużyłło W, Kępka C (2022). Efficacy and safety of coronary computed tomography angiography in patients with a high clinical likelihood of obstructive coronary artery disease. Kardiol Pol.

[36] Chang HJ, Lin FY, Gebow D, An HY, Andreini D, Bathina R, Baggiano A, Beltrama V, Cerci R, Choi E, Choi J, Choi S, Chung N, Cole J, Doh J, Ha S, Her A, Kepka C, Kim J, Kim J, Kim S, Kim W, Pontone G, Valeti U, Villines TC, Lu Y, Kumar A, Cho I, Danad I, Han D, Heo R, Lee S, Lee JH, Park H, Sung J, Leflang D, Zullo J, Shaw LJ, Min JK (2019). Selective referral using CCTA versus direct referral for individuals referred to invasive coronary angiography for suspected CAD: a randomized, controlled, open-label trial. JACC Cardiovasc Imaging.

