# Supplementary material for: Coronary Computed Tomographic Angiography to Optimize the Diagnostic Yield of Invasive Angiography for Low-Risk Patients Screened With Artificial Intelligence: Protocol for the CarDIA-AI Randomized Controlled Trial
Source: JMIR Res Protoc. 2025 May 21;14:e71726. doi: 10.2196/71726 (PMC12138305; doi:10.2196/71726)
Supplement: Multimedia Appendix 1 [file resprot_v14i1e71726_app1.docx]

**SPIRIT-AI Checklist: Recommended items to address in a protocol and related documents for clinical trials evaluating AI interventions**

| Section |  | SPIRIT 2013 Item | | SPIRIT-AI Item | | Addressed on Page No |
| --- | --- | --- | --- | --- | --- | --- |
| Administrative Information | | | | | | |
| Title | 1 | Descriptive title identifying the study design, population, interventions, and, if applicable, trial acronym | | SPIRIT-AI 1(i) Elaboration | Indicate that the intervention involves artificial intelligence / machine learning and specify the type of model. | 0 (title page), 1 |
|  |  |  |  | SPIRIT-AI 1(ii) Elaboration | Specify the intended use of the AI intervention. | 1 |
| Trial registration | 2a | Trial identifier and registry name. If not yet registered, name of intended registry | |  |  | 1,4 |
|  | 2b | All items from the World Health Organization Trial Registration Data Set | |  |  | Supplementary Materials* |
| Protocol version | 3 | Date and version identifier | |  |  | 4 |
| Funding | 4 | Sources and types of financial, material, and other support | |  |  | 14 |
| Roles and responsibilities | 5a | Names, affiliations, and roles of protocol contributors | |  |  | 0, 13-14 |
|  | 5b | Name and contact information for the trial sponsor |  |  |  | 14 |
|  | 5c | Role of study sponsor and funders, if any, in study design; collection, management, analysis, and interpretation of data; writing of the report; and the decision to submit the report for publication, including whether they will have ultimate authority over any of these activities |  |  |  | 14 |
|  | 5d | Composition, roles, and responsibilities of the coordinating centre, steering committee, endpoint adjudication committee, data management team, and other individuals or groups overseeing the trial, if applicable (see Item 21a for data monitoring committee) |  |  |  | 14-15 |
| Introduction | | | | | | |
| Background and rationale | 6a | Description of research question and justification for undertaking the trial, including summary of relevant studies (published and unpublished) examining benefits and harms for each intervention |  | SPIRIT-AI 6a (i) Extension | Explain the intended use of the AI intervention in the context of the clinical pathway, including its purpose and its intended users (e.g. healthcare professionals, patients, public). | 2, 3 |
|  |  |  |  | SPIRIT-AI 6a (ii) Extension | Describe any pre-existing evidence for the AI intervention. | 2 |
|  | 6b | Explanation for choice of comparators |  |  |  | 3 |
| Objectives | 7 | Specific objectives or hypotheses |  |  |  | 3 |
| Trial design | 8 | Description of trial design including type of trial (eg, parallel group, crossover, factorial, single group), allocation ratio, and framework (eg, superiority, equivalence, noninferiority, exploratory) |  |  |  | 4 |
| Methods: Participants, Interventions and Outcomes | | | | | | |
| Study setting | 9 | Description of study settings (eg, community clinic, academic hospital) and list of countries where data will be collected. Reference to where list of study sites can be obtained |  | SPIRIT-AI 9 Extension | Describe the onsite and offsite requirements needed to integrate the AI intervention into the trial setting. | 4 |
| Eligibility criteria | 10 | Inclusion and exclusion criteria for participants. If applicable, eligibility criteria for study centres and individuals who will perform the interventions (eg, surgeons, psychotherapists) |  | SPIRIT-AI 10 (i) Elaboration | State the inclusion and exclusion criteria at the level of participants. | 5 |
|  |  |  |  | SPIRIT-AI 10 (ii) Extension | State the inclusion and exclusion criteria at the level of the input data. | 5 |
| Interventions | 11a | Interventions for each group with sufficient detail to allow replication, including how and when they will be administered |  | SPIRIT-AI 11a (i) Extension | State which version of the AI algorithm will be used. | 6 |
|  |  |  |  | SPIRIT-AI 11a (ii) Extension | Specify the procedure for acquiring and selecting the input data for the AI intervention. | 6, 8 |
|  |  |  |  | SPIRIT-AI 11a (iii) Extension | Specify the procedure for assessing and handling poor quality or unavailable input data. | 6, 8 |
|  |  |  |  | SPIRIT-AI 11a (iv) Extension | Specify whether there is human-AI interaction in the handling of the input data, and what level of expertise is required for users. | 6, 8 |
|  |  |  |  | SPIRIT-AI 11a (v) Extension | Specify the output of the AI intervention. | 6 |
|  |  |  |  | SPIRIT-AI 11a (vi) Extension | Explain the procedure for how the AI intervention’s output will contribute to decision-making or other elements of clinical practice. | 6 |
|  | 11b | Criteria for discontinuing or modifying allocated interventions for a given trial participant (eg, drug dose change in response to harms, participant request, or improving/worsening disease) |  |  |  | 6 |
|  | 11c | Strategies to improve adherence to intervention protocols, and any procedures for monitoring adherence (eg, drug tablet return, laboratory tests) |  |  |  | 6 |
|  | 11d | Relevant concomitant care and interventions that are permitted or prohibited during the trial |  |  |  | Supplementary Materials* |
| Outcomes | 12 | Primary, secondary, and other outcomes, including the specific measurement variable (eg, systolic blood pressure), analysis metric (eg, change from baseline, final value, time to event), method of aggregation (eg, median, proportion), and time point for each outcome. Explanation of the clinical relevance of chosen efficacy and harm outcomes is strongly recommended |  |  |  | 8 |
| Participant timeline | 13 | Time schedule of enrolment, interventions (including any run-ins and washouts), assessments, and visits for participants. A schematic diagram is highly recommended (see Figure) |  |  |  | 7 |
| Sample size | 14 | Estimated number of participants needed to achieve study objectives and how it was determined, including clinical and statistical assumptions supporting any sample size calculations |  |  |  | 4, 5 |
| Recruitment | 15 | Strategies for achieving adequate participant enrolment to reach target sample size |  |  |  | 5 |
| Methods: Assignment of Interventions (For Controlled Trials) | | | | | | |
| Sequence generation | 16A | Method of generating the allocation sequence (eg, computer-generated random numbers), and list of any factors for stratification. To reduce predictability of a random sequence, details of any planned restriction (eg, blocking) should be provided in a separate document that is unavailable to those who enrol participants or assign interventions |  |  |  | 5 |
| Allocation concealment mechanism | 16b | Mechanism of implementing the allocation sequence (eg, central telephone; sequentially numbered, opaque, sealed envelopes), describing any steps to conceal the sequence until interventions are assigned |  |  |  | 5 |
| Implementation | 16c | Who will generate the allocation sequence, who will enrol participants, and who will assign participants to interventions |  |  |  | 5 |
| Blinding (masking) | 17a | Who will be blinded after assignment to interventions (eg, trial participants, care providers, outcome assessors, data analysts), and how |  |  |  | 6 |
|  | 17b | If blinded, circumstances under which unblinding is permissible, and procedure for revealing a participant’s allocated intervention during the trial |  |  |  | N/A |
| Methods: Data Collection, Management, And Analysis | | | | | | |
| Data collection methods | 18a | Plans for assessment and collection of outcome, baseline, and other trial data, including any related processes to promote data quality (eg, duplicate measurements, training of assessors) and a description of study instruments (eg, questionnaires, laboratory tests) along with their reliability and validity, if known. Reference to where data collection forms can be found, if not in the protocol |  |  |  | 8, 9 |
|  | 18b | Plans to promote participant retention and complete follow-up, including list of any outcome data to be collected for participants who discontinue or deviate from intervention protocols |  |  |  | 9 |
| Data management | 19 | Plans for data entry, coding, security, and storage, including any related processes to promote data quality (eg, double data entry; range checks for data values). Reference to where details of data management procedures can be found, if not in the protocol |  |  |  | 9, Supplementary File 4 |
| Statistical methods | 20a | Statistical methods for analysing primary and secondary outcomes. Reference to where other details of the statistical analysis plan can be found, if not in the protocol |  |  |  | 10 |
|  | 20b | Methods for any additional analyses (eg, subgroup and adjusted analyses) |  |  |  | 10, 11 |
|  | 20c | Definition of analysis population relating to protocol non-adherence (eg, as randomised analysis), and any statistical methods to handle missing data (eg, multiple imputation) |  |  |  | 10 |
| Methods: Monitoring | | | | | | |
| Data monitoring | 21a | Composition of data monitoring committee (DMC); summary of its role and reporting structure; statement of whether it is independent from the sponsor and competing interests; and reference to where further details about its charter can be found, if not in the protocol. Alternatively, an explanation of why a DMC is not needed |  |  |  | Supplementary Materials* |
|  | 21b | Description of any interim analyses and stopping guidelines, including who will have access to these interim results and make the final decision to terminate the trial |  |  |  | 10 |
| Harms | 22 | Plans for collecting, assessing, reporting, and managing solicited and spontaneously reported adverse events and other unintended effects of trial interventions or trial conduct |  | SPIRIT-AI 22 Extension | Specify any plans to identify and analyse performance errors. If there are no plans for this, explain why not. | 11 |
| Auditing | 23 | Frequency and procedures for auditing trial conduct, if any, and whether the process will be independent from investigators and the sponsor |  |  |  | 11 |
| Ethics and Dissemination | | | | | | |
| Research ethics approval | 24 | Plans for seeking research ethics committee/institutional review board (REC/IRB) approval |  |  |  | 4, 11 |
| Protocol amendments | 25 | Plans for communicating important protocol modifications (eg, changes to eligibility criteria, outcomes, analyses) to relevant parties (eg, investigators, REC/IRBs, trial participants, trial registries, journals, regulators) |  |  |  | 15 |
| Consent or assent | 26a | Who will obtain informed consent or assent from potential trial participants or authorised surrogates, and how (see Item 32) |  |  |  | 4, 11-12 |
|  | 26b | Additional consent provisions for collection and use of participant data and biological specimens in ancillary studies, if applicable |  |  |  | N/A |
| Confidentiality | 27 | How personal information about potential and enrolled participants will be collected, shared, and maintained in order to protect confidentiality before, during, and after the trial |  |  |  | 12 |
| Declaration of interests | 28 | Financial and other competing interests for principal investigators for the overall trial and each study site |  |  |  | 15 |
| Access to data | 29 | Statement of who will have access to the final trial dataset, and disclosure of contractual agreements that limit such access for investigators |  | SPIRIT-AI 29 Extension | State whether and how the AI intervention and/or its code can be accessed, including any restrictions to access or re-use. | 6, 15 |
| Ancillary and post-trial care | 30 | Provisions, if any, for ancillary and post-trial care, and for compensation to those who suffer harm from trial participation |  |  |  | Supplementary Materials* |
| Dissemination policy | 31a | Plans for investigators and sponsor to communicate trial results to participants, healthcare professionals, the public, and other relevant groups (eg, via publication, reporting in results databases, or other data sharing arrangements), including any publication restrictions |  |  |  | Supplementary Materials* |
|  | 31b | Authorship eligibility guidelines and any intended use of professional writers |  |  |  | Supplementary Materials* |
|  | 31c | Plans, if any, for granting public access to the full protocol, participant-level dataset, and statistical code |  |  |  | Supplementary Materials* |
| Appendices | | | | | | |
| Informed consent materials | 32 | Model consent form and other related documentation given to participants and authorised surrogates |  |  |  | Supplementary File 2 |
| Biological specimens | 33 | Plans for collection, laboratory evaluation, and storage of biological specimens for genetic or molecular analysis in the current trial and for future use in ancillary studies, if applicable |  |  |  | N/A |

*Item 2b: See page 6 of this document.

*Item 11d: In line with the pragmatic nature of this trial, no restrictions with respect to concomitant care and interventions will be made.

*Item 21a: A formal DMC will not be implemented as the trial intervention poses minimal risks to the participants. However, a data quality committee will be formed to review the cross-referral process between ICA and CCTA, to ensure that indicated procedures are booked and performed, and to monitor model performance.

*Item 30: As the intervention is minimal-risk, no provisions for ancillary or post-trial care are planned.

*Item 31a: Study leads, co‐leads and co‐investigators intend to publish in peer‐reviewed journals and make conference presentations. The results will be disseminated regardless of the magnitude or direction of effect.

*Item 31b: Research team members intend to prepare manuscripts without the assistance of professional writers. Publication of this protocol and any future related publications will follow ICMJE authorship guidelines.

*Item 31c: Individual participant data will be stored for future use by the internal research team only.

**SPIRIT-AI Item 2b: WHO Trial Registration Data Set**

| **Data category** | **Information** |
| --- | --- |
| Primary registry and trial identifying number | ClinicalTrails.gov NCT06648239 |
| Date of registration in primary registry | 18 October 2024 |
| Secondary identifying numbers | Hamilton Integrated Research Ethics Board (HiREB) #17103 |
| Source(s) of monetary or material support | Hamilton Academic Health Sciences Organization (HAHSO), Population Health Research Institute (PHRI) |
| Primary sponsor | Hamilton Health Sciences Corporation |
| Secondary sponsor(s) | N/A |
| Contact for public queries | petchj@hhsc.ca |
| Contact for scientific queries | petchj@hhsc.ca |
| Public title | CCTA to Optimize Diagnostic Yield of Invasive Angiography with AI (CarDIA-AI) |
| Scientific title | Coronary computed tomographic angiography to optimize the Diagnostic yield of Invasive Angiography for low-risk patients screened with Artificial Intelligence |
| Countries of recruitment | Canada |
| Health condition(s) or problem(s) studied | Coronary artery disease |
| Intervention(s) | Experimental: Centralized triage with AI-based screening for obstructive CAD  Comparator: Usual care |
| Key inclusion and exclusion criteria | Inclusion criteria: Patients are eligible if they (1) are ≥18 years of age; (2) are referred for non-urgent (elective) outpatient ICA; (3) have an indication for ICA that includes ‘Rule out CAD’, ‘Cardiomyopathy’, ‘Stable CAD’ or ‘Stable Angina’; and (4) are able to provide informed consent in English |
|  | Exclusion criteria: Patients will be excluded if they (1) have received a prior high-quality CCTA within the last five years; (2) have atrial fibrillation; (3) have known severe renal dysfunction (GFR <35); (4) have planned non-coronary cardiac surgery; (5) have any prior obstructive CAD, acute coronary syndrome (ACS), percutaneous coronary intervention (PCI), or coronary artery bypass graft (CABG); or (6) have known severe coronary artery calcification (calcium score >1000) |
| Study type | Interventional |
|  | Allocation: randomized; Intervention model: parallel assignment; Masking: open label |
|  | Primary purpose: screening |
| Date of first enrollment | October 2024 (anticipated) |
| Target sample size | 252 |
| Recruitment status | Not yet recruiting |
| Primary outcome(s) | Rate of normal or non-obstructive CAD diagnosed via ICA |
| Key secondary outcomes | 1) Number of angiograms avoided; 2) deviation from management recommendations following CCTA (i.e., angiograms performed when not recommended); 3) diagnostic yield of ICA; 4) number of low-quality CCTAs; 5) difference in the rate of normal/non-obstructive CAD diagnosed through ICA between males and females; and 6) difference in the rate of normal/non-obstructive CAD diagnosed through ICA between sites |
