# Supplementary material for: Coronary Computed Tomographic Angiography to Optimize the Diagnostic Yield of Invasive Angiography for Low-Risk Patients Screened With Artificial Intelligence: Protocol for the CarDIA-AI Randomized Controlled Trial
Source: JMIR Res Protoc. 2025 May 21;14:e71726. doi: 10.2196/71726 (PMC12138305; doi:10.2196/71726)
Supplement: Multimedia Appendix 2 [file resprot_v14i1e71726_app2.docx]

**Patient Information Sheet and Consent Form**

**Title of Study:** Coronary computed tomographic angiography to optimize Diagnostic yield of Invasive Angiography for low-risk patients screened with Artificial Intelligence (CarDIA-AI)

**Principal Investigators:** Dr. Jon-David Schwalm, Dr. Jeremy Petch

**Co-investigators:** Dr. Tej Sheth, Dr. Natalia Pinilla, Dr. Madhu Natarajan

**Location:** Hamilton Health Sciences and Niagara Health System

**Funding Source:** Population Health Research Institute (PHRI) and Hamilton Academic Health Sciences Organization (HAHSO)

**Research Ethics Approval:** Hamilton Integrated Research Ethics Board (HiREB)

We would like to invite you to take part in our research study. Before you decide, it is important that you understand why the research is being done and what it would involve for you. Please take time to read this information, and discuss it with others if you wish. If there is anything that is not clear, or if you would like more information, please ask us.

**What is the purpose?**

This purpose of the CarDIA-AI study is to identify patients at a low risk for Coronary Artery Disease (CAD) through the use of a new risk assessment tool and centralized triage process to help facilitate a test called Coronary Computed Tomographic Angiography (CCTA) instead of a more invasive and higher risk test (Invasive Coronary Angiography). Our hope is that this new approach can help every patient get the diagnostic test that is best suited for them.

**Why have I been invited?**

We are inviting you to take part in the study because you have been referred to our Heart Investigation Unit to undergo invasive coronary angiography to determine whether you have blockages in the arteries of your heart. We are seeking 252 patients with suspected blockages in the arteries of the heart to take part in our study.

**Do I have to take part?**

No. Participation in this research is voluntary and it is important for you to know that you can choose not to take part in this study. Your decision to participate will not affect your current and future medical care. You can discuss alternative options with your doctor. If you choose to participate, you can withdraw from the research study at any time before the study closes and without having to provide any reason.

**What will happen to me if I decide to take part?**

If you choose to take part in the study, you will be randomly selected into one of two groups. The first group (the control) will receive an invasive coronary angiogram as they normally would. The second group (the experimental) will have details of their medical history, already included on your referral form (age, sex, history of heart disease, etc.) entered into our risk assessment tool. The risk assessment tool identifies patients who are good candidates for a CCTA scan, and which patients are good candidates for an invasive angiogram. Patients in the experimental group who are identified as good candidates for an invasive angiogram will be booked for the procedure by our HIU team, just like patients in the control group. Patients identified as good candidates for a CCTA scan will have the scan booked by research staff.

A CCTA scan is a medical imaging test that helps your doctor see the blood vessels that supply your heart. During this test, a special dye is injected into your bloodstream, and a CT scanner takes detailed pictures of your heart and blood vessels. The images can help your doctor identify blockages or other issues that may affect your heart health. Sometimes we use a medication to adjust your heart rate to get the best pictures.

What to expect:

- You will lie on a table that slides into the CT scanner.
- A contrast dye will be injected into a vein in your arm to make your blood vessels more visible on the images.
- The scanner will take a series of X-ray images as it moves around your chest.
- The procedure typically takes about 30 minutes.

**Are there any disadvantages or risks from taking part?**

We have completed a pilot study of this centralized triage process, and patients who received CCTA scans did not report any discomfort, delays, or other concerns with the experience. All invasive angiograms and CCTA scans will be completed within Ontario’s recommended time frames, so participation in this study will not result in any delays to your medical care. CCTA takes less time than an invasive angiogram to complete, carries less overall risk and is as accurate for identifying blockages in the major arteries of the heart as an invasive angiogram. The results of the test will help your doctor tailor your medications to improve your heart health.

There are some risks associated with CCTA. CCTA scans involve a higher dose of radiation than invasive angiograms, but on balance are considered safer because they are non-invasive. While the overall radiation risk is generally low, repeated exposure can increase the risk of cancer. In rare cases, some people may have an allergic reaction to the contrast dye or experience a change in kidney function, especially in people with pre-existing kidney conditions. The risks related to the contrast dye and kidney function can also occur with invasive angiograms. When a CCTA is deemed appropriate by your doctor, the benefits of the scan to guide health care decisions are greater than the risks.

Most patients who get a CCTA scan will be diagnosed with no or non-significant blockages in the arteries of the heart but for some patients, the CCTA scan demonstrates a severe blockage in one or more arteries of the heart. In these cases, our HIU team will arrange for an invasive coronary angiogram. Based on previous studies we expect this to happen in less than 3 out of 10 people undergoing this test.

If you suffer an injury from participation in this study, medical care will be made available to you by your study doctor, or you will be referred for appropriate medical care.

**What are the possible benefits of taking part?**

We cannot promise any personal benefits to you from your participation in this study. However, possible benefits include receiving a noninvasive CCTA instead of an invasive angiogram. Your participation may help other people with heart disease in the future.

**Will there be any costs to me?**

Participation in this study will not involve any additional costs to you.

**Will I be paid to participate?**

You will not be paid for taking part in this study.

**Will my cardiologist be informed of my participation?**

Yes, we will contact your cardiologist with the results of whichever test you receive.

**Will my taking part in the study be kept confidential?**

Yes. All identifying information collected for this study will be stored on secure servers at Hamilton Health Sciences and Niagara Health and will only be accessed by members of the study team. It is possible that representatives of the Hamilton Integrated Research Ethics Board (HiREB), this institution, and affiliated sites may consult your original (identifiable) research data and medical records to check that the information collected for the study is correct and follows proper laws and guidelines. Data collected for the study will be de-identified prior to being analyzed. Your identifiable data will not be shared with anyone except with your consent or as required by law. If the results of the study are published, your name will not be used and no information that discloses your identity will be released or published without your specific consent to the disclosure.

**Who do I contact if I have questions, concerns, or want to withdraw?**

If you have any questions, concerns or wish to withdraw, you can contact the principal investigator, Dr. Schwalm at [schwalj@mcmaster.ca](mailto:schwalj@mcmaster.ca) or 905-577-1423. You may choose to withdraw up until the study is closed and before the data is analyzed.

**Signature of Research Participant**

| **Verbal Consent:**  *This section will be completed if the participant is providing verbal informed consent*  The participant has read the consent form, had an opportunity to ask question and those questions have been answered. The participant verbally agreed to participation in the study. The participant will receive a copy of the informed consent form including this completed signature page.  _________________________ ______________________  PRINTED NAME of Participant Date consent was provided    __________________________ ______________________ ______________________  Signature of Person Conducting PRINTED NAME & ROLE Date  the Consent Discussion |
| --- |

| **Written Consent**  *This section will be completed if the participant is providing written informed consent*  I have read the preceding information thoroughly. I have had an opportunity to ask questions and all of my questions have been answered to my satisfaction. I agree to participation in the study. I understand that I will receive a signed copy of this form.   \| PRINTED NAME of Participant \| Signature \| Date \| \| --- \| --- \| --- \|     __________________________ ____________________ ______________  Signature of Person Conducting PRINTED NAME & ROLE Date  the Consent Discussion |
| --- | --- | --- | --- |

This study has been reviewed by the Hamilton Integrated Research Ethics Board (HiREB). The HiREB is responsible for ensuring that participants are informed of the risks associated with the research, and that participants are free to decide if participation is right for them. If you have any questions about your rights as a research participant, please call the Office of the Chair, Hamilton Integrated Research Ethics Board at 905.521.2100 x 42013.
