# Supplementary material for: Coronary Computed Tomographic Angiography to Optimize the Diagnostic Yield of Invasive Angiography for Low-Risk Patients Screened With Artificial Intelligence: Protocol for the CarDIA-AI Randomized Controlled Trial
Source: JMIR Res Protoc. 2025 May 21;14:e71726. doi: 10.2196/71726 (PMC12138305; doi:10.2196/71726)
Supplement: Multimedia Appendix 3 [file resprot_v14i1e71726_app3.docx]

## Table S1. CCTA Results and Clinical Recommendations.

| **CCTA Result** | **Clinical Recommendations** |
| --- | --- |
| **Non-diagnostic scan** and/or one or more non-evaluable coronary segments | Invasive angiogram will be arranged.* |
| **Normal** | Primary risk factor modification suggested. Invasive angiogram not recommended. |
| **Mild CAD:** <50% stenosis in any major epicardial coronary vessel (>2 mm in diameter). | Secondary risk factor modification and clinical follow-up. Invasive angiogram not recommended. |
| **Borderline CAD:** 50–69% stenosis in any major epicardial vessel (>2 mm in diameter) OR stenosis of >50% that cannot be quantified more precisely due to calcification or motion artifact. | Secondary risk factor modification and clinical follow-up.  Invasive angiogram will be arranged.* |
| **Significant CAD:** ≥70% stenosis in any major epicardial coronary vessels (>2 mm in diameter) or >50% stenosis in the left main. | Secondary risk factor modification and clinical follow-up.  Invasive angiogram will be arranged.* |

*Exceptions may be made if the lesion involves a clearly non-prognostic territory and/or there are minimal or no symptoms.

CAD = coronary artery disease, CCTA = coronary computed tomographic angiography

##
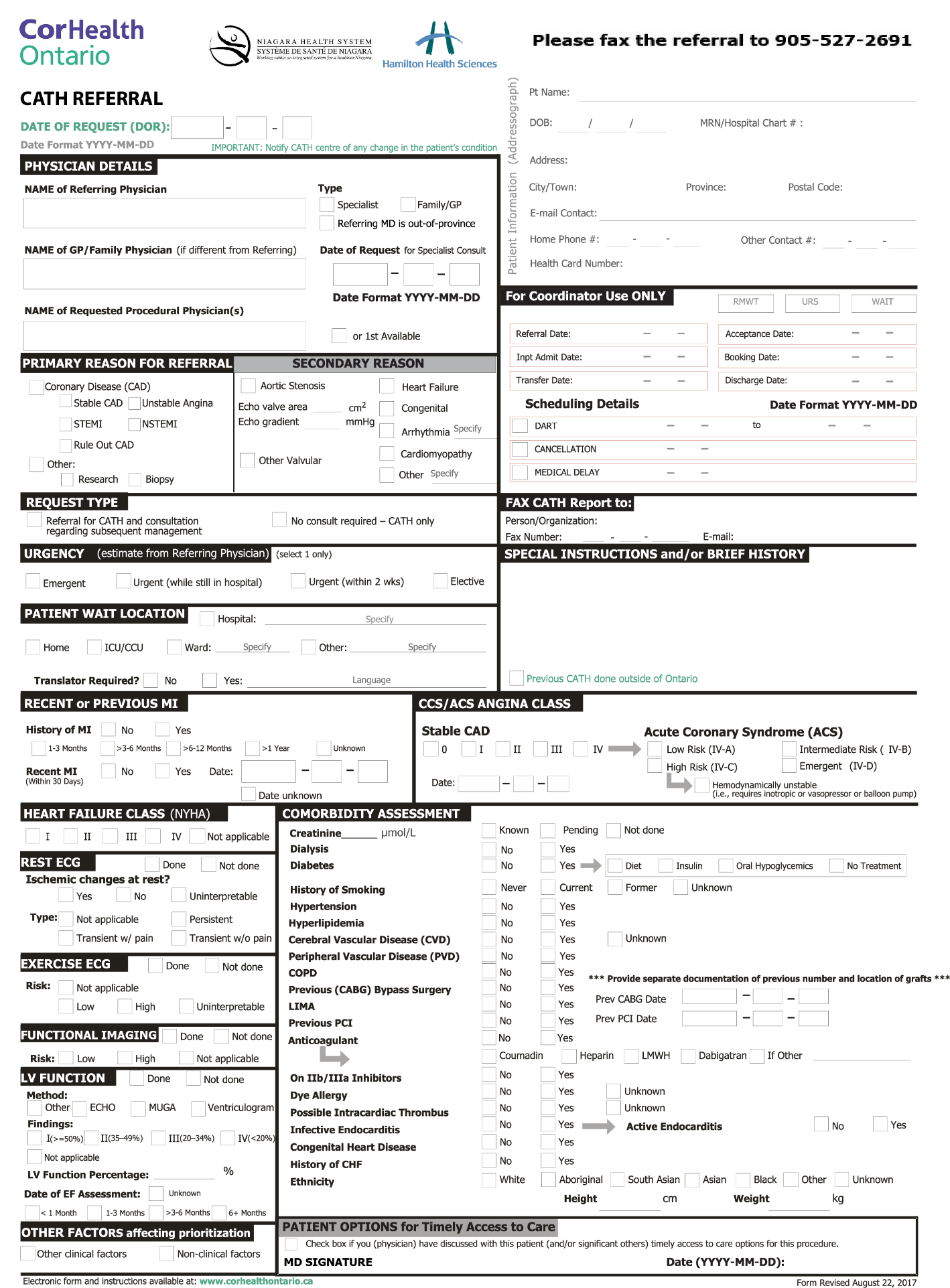
Figure S1. ICA referral form used by the two trial centres.

##
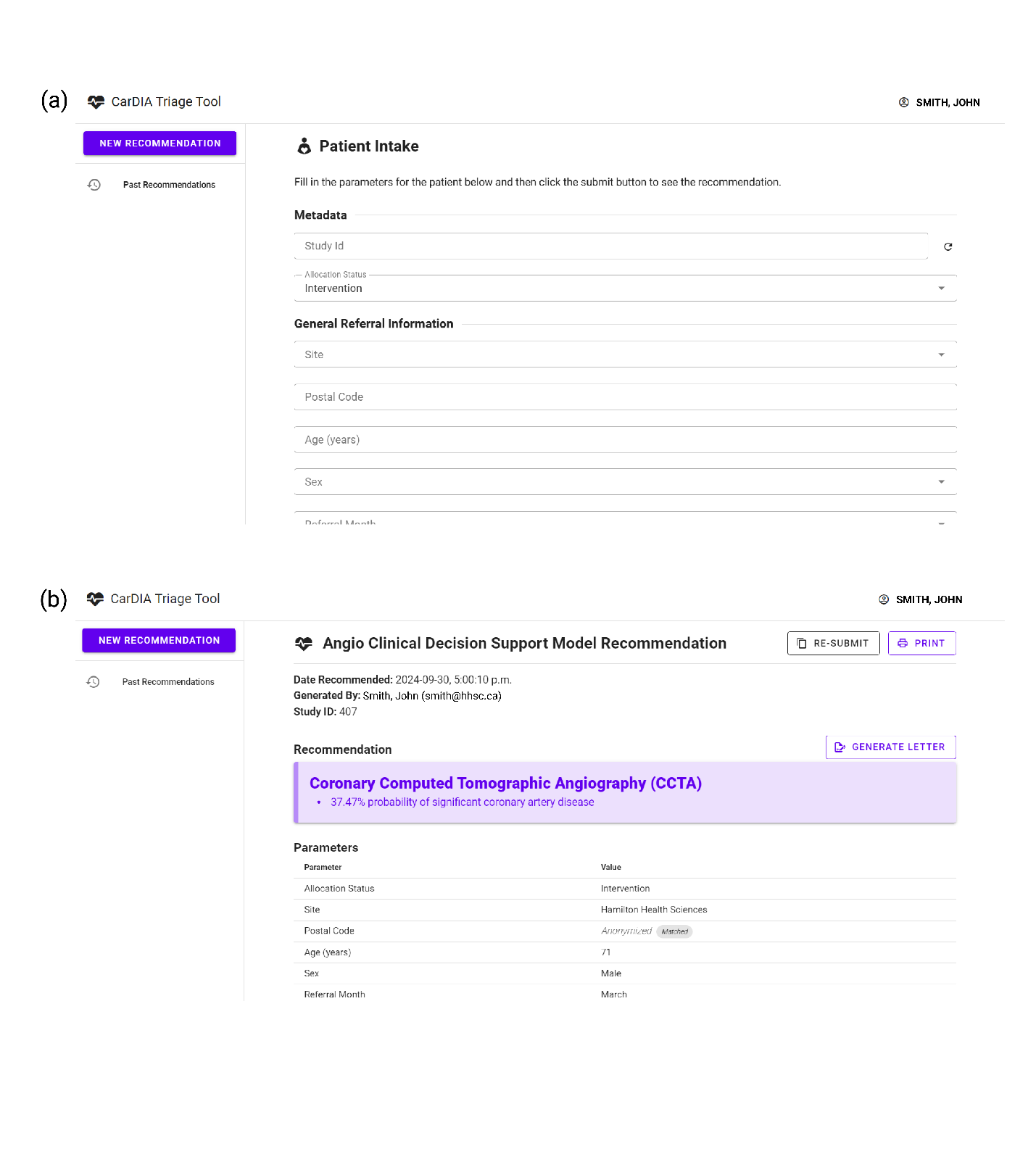


## Figure S2. User interface of the CarDIA-AI decision support tool. (a) Section of the data entry form of the decision support tool. (b) Recommendation screen of the decision support tool.
