# Supplementary material for: Coronary Computed Tomographic Angiography to Optimize the Diagnostic Yield of Invasive Angiography for Low-Risk Patients Screened With Artificial Intelligence: Protocol for the CarDIA-AI Randomized Controlled Trial
Source: JMIR Res Protoc. 2025 May 21;14:e71726. doi: 10.2196/71726 (PMC12138305; doi:10.2196/71726)
Supplement: Multimedia Appendix 4 [file resprot_v14i1e71726_app4.docx]

The machine learning-based clinical decision support algorithm is a Light Gradient Boosting Machine (LightGBM) model that predicts the probability of obstructive CAD, defined as ≥70% stenosis in major epicardial vessels of >2 mm in diameter or ≥50% stenosis of the left main artery, based on variables included on catheterization referral forms. Specifically, the predictors are demographic characteristics (age, sex), patient referral information (referral month, primary reason for referral, primary reason for referral–type, referring physician’s estimate of urgency, referring physician’s CPSO number, family physician status, request for first available procedural physician, need for translator), anthropometric measures (height, weight, BMI), clinical symptoms and risk factors (creatinine status, creatinine measurement, dialysis, diabetes, hypertension, hyperlipidemia, CVD, PVD, COPD, anticoagulant use, dye allergy, possible intracardiac thrombus, infective endocarditis, congenital heart disease, history of CHF), medical history (history of MI, recent MI, CCS/ACS class, NYHA class, ischemic changes at rest, exercise ECG risk category, functional imaging risk category, LV function evaluation method, LV function grade), and history of smoking. We were also able to include socioeconomic variables as a predictor by linking to the 2016 Ontario Marginalization Index using postal codes of patient residence. The Ontario Marginalization Index is a validated data tool that combines a wide range of demographic indicators to characterize 4 dimensions of marginalization: residential instability, material deprivation, ethnic concentration, and dependency.
