# Supplementary material for: Coronary Computed Tomographic Angiography to Optimize the Diagnostic Yield of Invasive Angiography for Low-Risk Patients Screened With Artificial Intelligence: Protocol for the CarDIA-AI Randomized Controlled Trial
Source: JMIR Res Protoc. 2025 May 21;14:e71726. doi: 10.2196/71726 (PMC12138305; doi:10.2196/71726)
Supplement: Multimedia Appendix 5 [file resprot_v14i1e71726_app5.docx]

## **General Best Practices**

- If fields are blank, leave blank for imputation – unless it is a comorbidity, then search for it in Epic and assume they do not have it if no mention
- If the form conflicts with letter regarding the presence of comorbidities, take the letter as correct
- If there was any information that was not readily available in the form/letter/note (i.e., you had to search for it in Epic), make note of where you found it in the “Other Comments” column of the spreadsheet

## **Tips for Filling Form Fields**

#### **Postal Code**

- Can be found in Chart Review > SnapShot

#### **Referral Month**

- This is the date that the clinic first received the referral
- From CorHealth Data Dictionary: “Regardless of the form of communication of a referral, this is intended to represent the **date the referral is received at the service location**, not the date the referral is assessed by the service location. In the case of a fax, this is the date stamped by the fax machine. In the case of an email, this is the date on the email.”

#### **Referring Physician CSPO Number**

- Name of the physician will be in the paper/Epic form as well as in the letter
- Use the CSPO doctor search tool to find their CSPO number: [CPSO - Find a Doctor](https://doctors.cpso.on.ca/?search=general)
  - Once entered in the tool, it will usually appear in the dropdown menu (if not, it is because this doctor’s referrals were not used to train the model – don’t worry about this)

#### **First Available Procedural Physician**

- Leave blank for imputation if not checked on form
- For Epic referrals: On the left sidebar there is usually a ‘Next Available HIU physician’ or something to that effect

#### **Family Physician**

- If the patient has a family physician, this will be listed in the left sidebar in Epic and on paper referral forms
  - Select “Yes” if the patient has a family physician and the physician’s name is on the form/in Epic
  - Select “No” if the patient does not have a family physician (this will usually be mentioned in the letter; the field in the Epic sidebar will also say “None”)
  - Select “Unknown” if the patient has a family physician, but the name of the physician is missing

#### **Primary Reason for Referral**

- This is listed in the paper/Epic referral form
- If a primary reason is given but no specific reason type, input the primary reason and leave the reason type blank for imputation
- If this is missing from the form, it is sometimes mentioned in the “Brief History” box in the paper referral or in the letter/note
  - Only use this option if the referral reason is explicitly stated as “CAD” or “rule out CAD”; otherwise, do not make assumptions and leave blank for imputation
- If completely missing, leave blank for imputation

#### **Estimate of Urgency**

- Eligible patients will have:
  - Low Urgency
    - “Elective” on the paper form
    - “Elective” on the Epic form
  - Moderate Urgency:
    - “Urgent (within 2 wks)” on the paper form
    - “Urgent (HIU/EP only)” on the Epic form, but **only if** Wait location = “Home” and Reason Type = “Elective, stable CAD” or “Rule out CAD”
- There is no situation where you would select High or Critical Urgency as these patients are ineligible
  - On the forms, “Urgent (while still in hospital)” and “Emergent” indicate ineligibility based on urgency
  - If the physician has entered High or Critical Urgency but Omar has confirmed that it is a mistake, leave blank for imputation
- If missing, leave blank for imputation

#### **Translator Required?**

- For all RCT participants, this should be “No”
  - Patients who require a translator are not eligible for the study

#### **CCS/ACS Class & NYHA**

- For NYHA, look up classes to find corresponding numbers:
  - 1 = No symptoms with ordinary physical activity
  - 2 = Symptoms with ordinary activity. Slight limitations
  - 3 = Symptoms with less than ordinary activity. Marked limitations
  - 4 = Symptoms with any physical activity or even at rest
- If a range of classes is given, leave blank for imputation
- If missing, leave blank for imputation

#### **Ischemic Change Type**

- If Rest ECG = Not Done, select **Unknown**
- If Rest ECG = Done, and Ischemic Changes at Rest = No, select **Not applicable**
- If Rest ECG = Done, and Ischemic Changes at Rest = Uninterpretable, select **Unknown**
- If Rest ECG = Done, but results are unclear (e.g., the physician did not specify whether changes were observed or checked "yes" but didn't specify what kind of changes), select **Unknown**
- If Rest ECG = Done, but results are pending, select **Unknown**
- If missing, leave blank for imputation

#### **Exercise ECG**

- A.k.a. exercise stress test, stress test, stress electrocardiogram. Bruce protocol is usually used for this
  - Not to be confused with stress echocardiogram, nuclear stress test/MPI/MIBI using Bruce protocol
- If Exercise ECG = Not Done, select **Not applicable**
- If Exercise ECG = Done but risk is not completed on the form or is completed incorrectly, **leave blank** for imputation
- If Exercise ECG = Done, but Risk = Uninterpretable, select **Uninterpretable**
- If missing, leave blank for imputation

#### **Functional Imaging**

- A.k.a. nuclear stress test, nuclear/PET perfusion/myocardial perfusion imaging (MPI/MIBI), exercise/stress MUGA, stress echocardiography (from CorHealth)
  - Stress may be induced by exercise (e.g., Bruce protocol) or pharmacologically (e.g., medications such as dipyridamole/Persantine, adenosine, dobutamine/Dobutrex)
- If Functional Imaging = Not Done, select **Not applicable**
- If Functional Imaging = Done but risk is not completed on the form or is completed incorrectly (e.g., multiple options are selected), **leave blank** for imputation
- There is no situation where you would select “Unknown”
- If missing, leave blank for imputation

#### **LV Method/Function**

- Most common method: Echo
  - A.k.a. transthoracic echocardiogram (TTE), LVEF by Simpson’s method
- If the LV assessment was not completed, select LV Method = **Not Done** and LV Function = **Not applicable**
- If the LV assessment was completed but the results are not yet available, select LV Function = **Unknown**
- If the LV assessment was completed but no boxes are checked, select LV Function = **Unknown**
- If missing, leave blank for imputation

#### **Creatinine Description/Value**

- See tips above for finding the creatinine value
- If there are conflicting Cr values in the form and letter/note, look for the most recent value with a date attached, whether it is in the form/letter/Epic
- If the physician has indicated that results are “pending” or “not done”:
  - Search for any Cr values <2 years old within Epic – if a value is found, enter Creatinine Description = **Known** and enter this Cr value, making note of the date of this value and where it was found in the ‘Other Comments’ column
  - If no values are available in Epic, enter Creatinine Description = **Pending/Not Done** (based on what the physician has checked) and leave the Cr value field blank for imputation
- If no information is available, leave both fields blank for imputation
  - There is no situation where you would select Creatinine Description = Unknown

#### **History of Smoking**

- Almost always listed on paper/Epic forms and in letter/note
- CorHealth definition of “Current”: “Present use of any form of tobacco less than or equal to 30 days of referral date.”
- CorHealth definition of “Former”: “History of any form of tobacco use greater than 30 days of referral date.”
  - If the patient used tobacco very sparingly, they are still considered a former smoker (per CorHealth definition)
- Electronic cigarettes (vaping) and cannabis are not considered tobacco products (per CorHealth)
- If history unknown, select **Unknown**

#### **Hypertension**

- Frequently abbreviated as HTN
- If no mention in letter/note or elsewhere in Epic, assume they don’t have it

#### **Hyperlipidemia**

- Frequently abbreviated as HLP; also known as dyslipidemia/DLP
- “High cholesterol” also indicates hyperlipidemia
  - Don’t attempt to interpret bloodwork results
- If no mention in letter/note or elsewhere in Epic, assume they don’t have it

#### **Cerebral/Peripheral Vascular Disease (CVD/PVD)**

- This is usually mentioned explicitly in the letter/note
- PVD is also known as peripheral arterial disease (PAD)
- For CVD: The data dictionary defines CVD as “any history of stroke, transient ischemic attack (TIA), previous carotid endarterectomy/stent or any known carotid stenosis > = 70%”
  - If physician mentions possible history of CVD/any of the above, input **Unknown**
- If no mention in letter/note or elsewhere in Epic, assume they don’t have it

#### **COPD**

- If no mention in letter/note or elsewhere in Epic, assume they don’t have it

#### **Anticoagulant**

- This is on the paper and Epic referral forms
- Look in the medication list for anticoagulants
- If the anticoagulant is not in the dropdown list, look up alternate names (generic/brand names)
  - If it is still not in the list, select a similar anticoagulant in consultation with a clinician
- Aspirin does not count as an anticoagulant
- If no mention in form/letter/note, assume they are not taking it

#### **Dye Allergy**

- This is in the paper form
- Allergies will be listed in the left sidebar of Epic
- Look for “contrast dye allergy” in the letter/note
- If no mention, assume they don’t have it

#### **Possible Intracardiac Thrombus/Infective Endocarditis/Congenital Heart Disease**

- If no mention in letter/note or elsewhere in Epic, assume they don’t have it

#### **History of Congestive Heart Failure (CHF)**

- A.k.a. heart failure (HF), heart failure with preserved/reduced ejection fraction (HFpEF/HFrEF)
- This is on the paper and Epic referral forms
- If no mention in letter/note or elsewhere in Epic, assume they don’t have it

#### **Weight/Height/BMI**

- Sometimes mentioned on paper form/letter/Epic note
  - In Epic, this may be in the free text or at the bottom, under “Additional Documentation”
- If not in form/letter/note:
  - Look in the Epic sidebar for height/weight measurements
  - Look within “Media” in Epic for studies from the same day (or <2 years prior) that mention weight/height
  - Search within Epic for recent notes/reports that mention patient weight/height (within <2 years)
  - If no data is available, leave blank
- If you did not find the information in the form/letter/note, make note of where you found it in the “Other Comments” column
- If there are conflicting height/weight values in the form and letter/note, look for the most recent value with a date attached, whether it is in the form/letter/Epic
- If BMI is not given but height and weight are available, use an online BMI calculator to calculate BMI
