# Supplementary material for: Coronary Computed Tomographic Angiography to Optimize the Diagnostic Yield of Invasive Angiography for Low-Risk Patients Screened With Artificial Intelligence: Protocol for the CarDIA-AI Randomized Controlled Trial
Source: JMIR Res Protoc. 2025 May 21;14:e71726. doi: 10.2196/71726 (PMC12138305; doi:10.2196/71726)
Supplement: Multimedia Appendix 6 [file resprot_v14i1e71726_app6.docx]

The interview and survey questions for the CarDIA-AI Implementation Study will be selected and adapted from the list of questions below. The development of these questions was informed by the updated Consolidated Framework for Implementation Research (CFIR), a determinant framework to explain barriers and facilitators to implementation effectiveness^1^.

- What is your understanding of the evidence supporting the implementation of the proposed AI-enabled triage pathway?
- Do you believe the pathway is supported by strong evidence demonstrating its effectiveness?
- How does the proposed pathway compare to current practice?
- Is the proposed process an improvement?
- Can the pathway be easily adapted to fit the specific needs and context of your site?
- How complex do you find the new pathway?
- Are there specific aspects that you think might be challenging to implement at your site?
- Would the current organization of tasks and responsibilities (between individuals and teams, including staffing levels) support the implementation/use of the pathway?
- How effectively are new initiatives communicated at your site?
- Do you believe that your existing workplace culture (i.e., values, beliefs and norms of individuals and teams) supports the successful use of the pathway?
- Do you believe that there is a need to change the current process for CAD screening?
- How well would the proposed pathway fit within your existing work processes and practices?
- How would the implementation of this new triage pathway compare to other ongoing activities or initiatives, in terms of priority?
- Are the necessary resources (e.g., funding, space, equipment) currently available to implement this pathway?
- What supports, in terms of training or education, would be helpful for new sites adopting this pathway?
- Do you believe you have the necessary knowledge and skills to effectively implement the pathway?
- What factors might affect your confidence in implementing the new triage pathway?
- Do you believe that implementing the new triage pathway aligns with your professional responsibilities?
- How motivated/committed do you feel towards fulfilling your role/responsibilities in implementing the pathway?
- Do you believe its implementation will be effective, in terms of reducing the rate of normal or non-obstructive CAD diagnosed through invasive coronary angiography?
- Do you believe that the implementation of this pathway will positively impact patient outcomes?
- What are some important considerations when developing the planning process for implementing the new triage pathway?
- Are there any particular stakeholders who should be involved in the implementation of this pathway at your site?
- How will/could the pathway and its implementation be monitored and evaluated, to ensure it meets the intended goals and outcomes?
- What aspects of the new triage pathway do you think might need to be customized for effective implementation at your site?
- How confident are you in your ability to implement the new triage pathway?
- How supportive would your colleagues be in implementing the new pathway?
- How do you feel about the implementation of the pathway?
- Does anything prevent you from ordering a CCTA in lower risk patients? If yes, What prevents you from ordering a CCTA in lower risk patients? (e.g., wait times, heart rate and beta blocker prescription, repeating studies if positive, calcium, etc.)
- Do you have any other comments about the AI-informed triage pathway or its implementation? Can you think of any other barriers or facilitators to its implementation that you have not already mentioned?

**Reference**

1. Damschroder, L.J., Reardon, C.M., Widerquist, M.A.O. *et al.* The updated Consolidated Framework for Implementation Research based on user feedback. *Implementation Sci* 17, 75 (2022).
