# Supplementary material for: Coronary Computed Tomographic Angiography to Optimize the Diagnostic Yield of Invasive Angiography for Low-Risk Patients Screened With Artificial Intelligence: Protocol for the CarDIA-AI Randomized Controlled Trial
Source: JMIR Res Protoc. 2025 May 21;14:e71726. doi: 10.2196/71726 (PMC12138305; doi:10.2196/71726)
Supplement: Multimedia Appendix 7 [file resprot_v14i1e71726_app7.docx]

Trial sponsor: Hamilton Health Sciences Corporation
Contact: Jeremy Petch

Address: 175 Longwood Road South, Suite 207, Hamilton, Ontario, Canada L8P 0A1

Email: petchj@hhsc.ca

**Steering Committee**

The Steering Committee will include the PIs (Jeremy Petch, JD Schwalm), Clinical Heads of Interventional Cardiology (Tej Sheth, Michael Tsang), Clinical Heads of CCTA/Radiology (Natalia Pinilla, Julian Dobranowski), Program Manager at PHRI (Tara McCready), Clinical Head of Cardiology (Adnan Hameed), Statistics (Shrikant Bangdiwala, Shuang Di), Knowledge Translation (Karen Mosleh), and CorHealth (Cathy Cattaruzza). The Steering Committee will aim to meet by phone monthly and approximately twice a year in person to review study progress (enrollment, adherence, dropouts, and data completeness and quality).

**Data Quality Committee**

The Data Quality Committee will comprise the PIs (Jeremy Petch, JD Schwalm), Safety Lead (Kevin Bainey), and the centralized triage staff. The Data Quality Committee will aim to conduct quarterly review, during the intervention period, of all eligible and screened participants. This review will focus on the cross-referral process between ICA and CCTA, to ensure that no participants are missed and that indicated testing/procedures are booked and performed as planned. Any gaps in the referral system identified, as well as concerns from administration, patients and/or referring physicians will be addressed. Model performance will also be monitored periodically.

Any modifications to the protocol that may impact the execution, scope, scientific validity, or ethical rigour of the trial will require a formal amendment to the protocol. Any amendments will be approved by the REB and Steering Committee prior to implementation. The principal investigators, statisticians, and delegates for sub-study analysis will have access to the final de-identified dataset, as approved by the Steering Committee and REB.
